# Engineered ubiquitin ligase PTB-U-box targets insulin/insulin-like growth factor receptor for degradation and coordinately inhibits cancer malignancy

**DOI:** 10.18632/oncotarget.2066

**Published:** 2014-06-06

**Authors:** Qinhao Wang, Yi Ru, Daixing Zhong, Jing Zhang, Libo Yao, Xia Li

**Affiliations:** ^1^ State Key Laboratory of Cancer Biology, Departments of Biochemistry and Molecular Biology, the Fourth Military Medical University, Xi’an, Shaanxi, China; ^2^ Department of Thoracic Surgery, Tangdu Hospital, the Fourth Military Medical University, Xi’an, Shaanxi, China; ^3^ Experiment Teaching Center, the Fourth Military Medical University, Xi’an, Shaanxi, China; ^4^ State Key Laboratory of Cancer Biology, Department of Biochemistry and Molecular Biology, the Fourth Military Medical University, Xi’an, Shaanxi, China

**Keywords:** IGF-1R, IR, cancer therapy, engineered ubiquitin ligase, protein degradation

## Abstract

The type 1 insulin-like growth factor receptor (IGF-1R) is a promising target for cancer therapy with antibodies and small molecule tyrosine kinase inhibitors (TKIs) which have been actively tested clinically. Evidences have demonstrated that insulin receptor (IR), which is implicated in tumorigenesis, conveys resistance to IGF-1R targeted therapy. This provided the compelling rationale for co-targeting IGF-1R and IR. Herein we have developed an approach to simultaneously down-regulate IGF-1R and IR in protein levels. By generating and screening several engineered ubiquitin ligases, we have identified that, PTB-U-box, which is composed of an IGF-1R/IR-binding domain and a functional E3 ubiquitin ligase domain, binds activated IGF-1R/IR and targets their ubiquitination and degradation. When ectopically expressed in HepG2 and HeLa cells, PTB-U-box inhibits cell proliferation and invasion, increases chemo-sensitivity, as well as interrupts glucose metabolism. Finally, intratumoral injection of adenovirus carrying PTB-U-box dramatically retards the growth of HepG2 xenograft. Therefore, well-designed engineered ubiquitin ligase represents an effective therapeutic strategy for the treatment of the cancers with co-expressed IGF-1R/IR.

## INTRODUCTION

Insulin and insulin-like growth factor (IGF) function as potent mitogen and metabolic modulators by binding to insulin and IGF-1 receptors, a subfamily of receptor tyrosine kinase (RTK) family, which in turn activate Akt and mitogen-activated protein kinase (MAPK) signaling networks [1, 2]. Growing experimental and clinical evidences demonstrated that insulin and IGF-1 receptor family are commonly expressed and have important roles in various neoplasia, rendering them as promising therapeutic targets [1, 2]. The drug candidates that target insulin and IGF-1 receptor family include anti-receptor antibodies, anti-ligand antibodies, receptor-specific tyrosine kinase inhibitors, and other agents with novel mechanisms [1, 3, 4]. Among them, the antibodies against IGF-1R have been actively developed and undertaken into clinical trials [1, 2, 4], but the most recent outcomes of Phase II and Phase III clinical trial were disappointing [5, 6]. Thus, it is important to re-consider current clinical trial programs and/or develop novel therapeutic strategies in this field.

Insulin receptor (IR) and Insulin-like growth factor I receptor (IGF-1R) are transmembrane proteins with similar structure, comprising of two extracellular α-subunits with the ligand-binding site and two transmembrane β-subunits with intracellular tyrosine kinase activity [7]. In addition, IR/IGF-1R “hybrid” receptor is formed by a half receptor from IR and a half receptor from IGF-1R [8]. Binding of insulin, IGF-1 or IGF-2 to the extracellular portion of the IR, IGF-1R or hybrid receptor stimulate the β-subunit tyrosine kinase activity and consequent phosphorylation of additional tyrosine residues, which in turn recruits insulin receptor substrates (IRS) and other adaptor proteins, allowing activation of the Akt and MAPK signaling pathways [9, 10]. Over-activation of IGF/IGF-1R and insulin/IR signaling pathway has been reported to promote several cancer hallmarks, including uncontrolled cell proliferation, migration, transformation, metastasis, angiogenesis and glycolysis [8-11]. Gene expression databases have revealed that most cancers express both the gene encoding the insulin receptor and the gene encoding the IGF-1R [12, 13]. Moreover, it is the case that when both receptors are expressed, “hybrid” receptors are always present on the cell surfaces [14, 15]. Importantly, it has been shown that insulin receptor conveys intrinsic resistance to IGF-1R targeted therapy [16-18]. Therefore, novel approaches that target both receptors may represent promising therapeutic strategies.

Covalent modification of the protein by ubiquitin, i.e., ubiquitination, can target the substrates for degradation in proteasome or lysosome [19, 20]. Ubiquitination is a three-step enzymatic reaction that is carried out by several enzymes: ubiquitin-activating enzyme (E1), ubiquitin conjugating protein (E2) and ubiquitin ligase (E3) [21]. E3s are responsible for transferring ubiquitin from E2 or E3 to the substrates they recognize. Thus, E3 determines the substrate specificities [22]. According to their functional domain, E3s can be divided into HECT-type E3s with a homologous to E6-AP COOH terminus (HECT) domain [23], RING-finger proteins [24] and U-box proteins [25]. The U-box protein CHIP (carboxy terminus of Hsc70 interacting protein) can bind to the molecular chaperone Hsc70/Hsp90 via its three tetratricopeptide repeats (TPRs) and mediates ubiquitination and subsequent degradation of the client proteins of Hsp90/Hsc70 [26]. Cbl, as a RING finger E3, functions as a dominant “activated protein tyrosine kinase (PTK)-selective” ubiquitin ligase by binding the activated PTK and promoting their degradation [27]. In this study, we sought to specifically decrease the protein levels of both IR and IGF-1R through enhancing their protein degradation. We created two artificial IR/IGF-1R-targeted ubiquitin ligases by fusing the U-box of CHIP or RING finger of Cbl with PTB (phosphotyrosine binding) domain of IRS-1 (insulin receptor substrate-1), a downstream adaptor that is recruited to the phosphorylated tyrosine residues of activated IR/IGF-1R through its PTB. We demonstrated that the engineered ubiquitin ligase PTB-U-box can promote the ubiquitination and degradation of IGF-1R and IR, and thus effectively inhibit *in vitro* and *in vivo* malignant behaviors of liver cancer HepG2 and cervical cancer HeLa cells that over-express IGF-1R and IR.

## RESULTS

### The engineered ubiquitin ligases specifically down-regulate IGF-1R and IR protein levels

**Figure 1:**
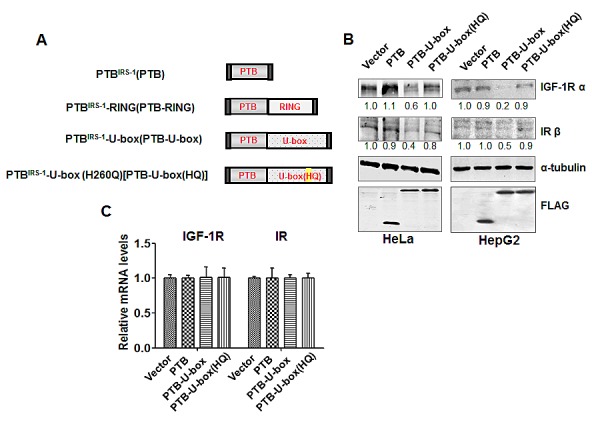
Generation of the engineered ubiquitin ligase A, Schematic representation of the engineered ubiquitin ligases. B, PTB-U-box promotes IGF-1R and IR down-regulation. HeLa and HepG2 cells were transiently transfected as indicated and analyzed by Western blotting. The bands intensity were quantified and normalized to the control. C, PTB-U-box does not change IGF-1R and IR transcription. Total RNA was isolated from the indicated transfectants and reverse-transcribed into cDNA. mRNA levels of IGF-1R (left) and IR (right) were measured by quantitative Real-time PCR.

Upon activation by insulin and IGF-1, the β-subunit tyrosine kinases of IR and IGF-1R mediate the phosphorylation of additional tyrosine residues, which will serve as the docking sites for the adaptor proteins such as insulin receptor substrates (IRS) [9, 10] (Supplementary figure 1A). Therefore, we generated the engineered ubiquitin ligases as shown in Fig.1A. PTB domain, which is derived from IRS-1, a primary adaptor of IGF-1R/IR signaling [9, 10], is responsible for recognizing and interacting with specific phospho-tyrosine residues of active receptors [28]. U-box domain from CHIP and RING finger domain from Cbl confer E3 ubiquitin ligase activity [25, 29]. PTB-U-box and PTB-RING were supposed to be sufficient for the functional E3 ligase activity and IGF-1R/IR targeting. PTB was created as the control that has only the binding domain. Additionally, PTB-U-box (HQ), which harbors a point mutation of H260Q that is known to disrupt the E3 activity of CHIP [30], was designed to serve as the counterpart of PTB-U-box without functional E3 activity. All of the constructs were cloned into pFLAG-CMV-4 to add the FLAG tag at the N-terminus.

To screen the effect of these recombinant constructs on IGF-1R, IGF-1R-encoding plasmid was transiently transfected into HEK293 cells together with empty vector, PTB, PTB-U-box or PTB-RING. Compared with empty vector and PTB, both PTB-U-box and PTB-RING are able to down-regulate IGF-1R protein in the presence of IGF-1, but PTB-U-box is more potent than PTB-RING (Supplementary figure 2). Thus, we mainly focused on PTB-U-box in this study.

We examined several cancer cell lines as for endogenous IGF-1R and IR levels, among which HepG2 and HeLa cells were chosen for the further study, because they express high levels of IGF-1R and IR and these receptors are constitutively activated when cultured in the serum-containing complete culture medium (Supplementary figure 1B). We found that IGF-1R and IR protein were significantly down-regulated in PTB-U-box transfected HepG2 cells and HeLa cells (Fig.1B). However, the cells transfected with vector, PTB and PTB-U-box(HQ) did not show significant decrease in IGF-1R and IR levels. Similar results were also obtained in PTB-U-box-transfected pancreatic cancer cell line PANC-1 (Supplementary figure 3). Meanwhile, IGF-1R and IR mRNA levels, analyzed by quantitative real-time PCR, were not significantly changed (Fig.1C), suggesting that their down-regulation occurred at post-transcriptional level. In addition, we examined the protein level of EGFR and Met, which were not designed to be targeted by our engineered ubiquitin ligase, and found that PTB-U-box did not affect these receptors (Supplementary figure 4). Together, these data indicated that PTB-U-box specifically decreases IGF-1R and IR protein levels and such effect depends on the functional U-box domain.

### The engineered ubiquitin ligase interacts with IGF-1R and IR and promotes their ubiquitination and degradation

**Figure 2:**
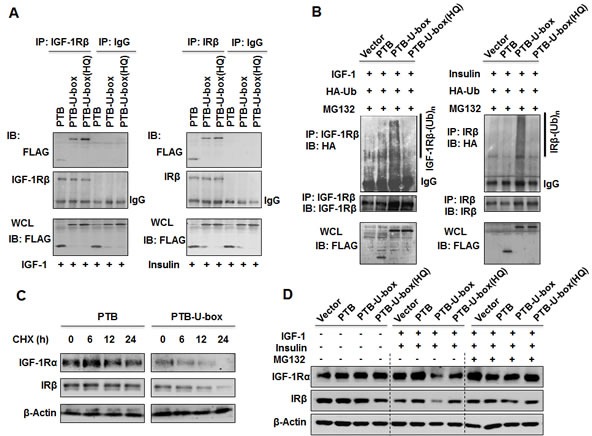
Engineered ubiquitin ligases promote the ubiquitination and degradation of IGF-1R and IR A, HeLa cells were transfected as indicated, treated with insulin or IGF-1 and lysed. Interaction between engineered ubiquitin ligase and IGF-1R or IR was determined by co-immunoprecipitation assay. B, HeLa cells were transfected with the indicated constructs together with pcDNA3.1(+)-3×HA-Ub, treated with MG-132 and insulin or IGF-1 as described in Material and Methods. IGF-1R and IR ubiquitination were assessed by *in vivo* ubiquitinatin assay. Whole cell lysates (WCL) were subjected to Western blotting with an anti-FLAG antibody. C, HeLa cells transfected as indicated were treated with CHX (50μg/ml) for 0, 6, 12 and 24 h. IGF-1R or IR protein stability was analyzed by Western blotting. D, HeLa cells transfected as indicated were treated with or without MG132 for 4 h and with or without specific ligands (IGF-1 and insulin) for 15min, IGF-1R or IR protein level was then determined by Western blotting.

Next, we examined whether PTB-U-box can interact with IGF-1R and IR and promote their ubiquitination. All FLAG-tagged constructs were transiently transfected into HeLa cells respectively, and co-immunoprecipitation assay and *in vivo* ubiquitination assay were performed. As expected, FLAG-tagged PTB-U-box and PTB-U-box(HQ) were co-immunoprecipitated with IGF-1R and IR as efficiently as PTB upon treatment with IGF-1 or insulin (Fig.2A). The result of *in vivo* ubiquitination assay clearly showed that over-expression of PTB-U-box was associated with an obvious increase in the ubiquitination of IGF-1R and IR, whereas the empty vector, deletion and H260Q mutation of U-box domain failed to enhance IGF-1R and IR ubiquitination (Fig.2B).

To further determine whether enhancement of IGF-1R and IR ubiquitination by PTB-U-box result in their degradation, we compared the stability of IGF-1R and IR in PTB and PTB-U-box expressing HeLa cells using cycloheximide (CHX) chase experiment. As shown in Fig.2C, expression of PTB-U-box markedly shortened the stability of IGF-1R and IR, suggesting that PTB-U-box caused ubiquitination of IGF-1R and IR resulted in their degradation. Moreover, IGF-1R and IR degradation mainly occurred in proteasome because MG-132 treatment inhibited downregulation of activated IGF-1R and IR (Fig.2D). Together, these results indicated that PTB-U-box could bind to, and promote ubiquitination of activated IGF-1R and IR, leading to their degradation in proteasome.

### PTB-U-box inhibits cancer cell proliferation and invasion

**Figure 3:**
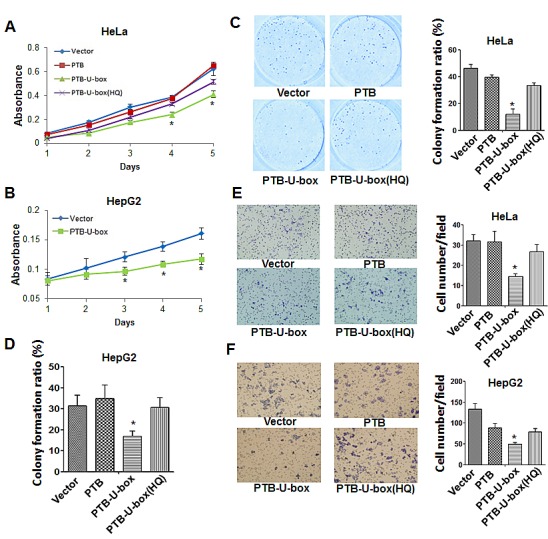
PTB-U-box inhibits cancer cell proliferation and invasion A and B, HeLa and HepG2 cells were transfected with the indicated constructs, and cell growth was measured by MTT assay for 5 days. C and D, HeLa and HepG2 cells transfected as indicated were seeded into six-well plates and cultured in the medium containing G418 to allow for colony formation. The colonies were stained and photographed and the colony formation ratio was assessed as described in Materials and Methods. E and F, HepG2 and HeLa cells that were transfected as in (C) were seeded onto matrigel-coated chamber for 24 h. The invasive cells were calculated in ten random fields (400×magnification). The histogram represents the quantification of invasive cells. In A-F, the results are represented as the mean±s.d. *P<0.05 for PTB-U-box *v.s.*vector.

It has been well documented that down-regulation of IGF-1R or insulin receptor inhibits cell proliferation, metastasis and *in vivo* tumor growth [31-33]. To investigate whether the engineered ubiquitin ligase could inhibit cancer cell growth, MTT assay was performed in HeLa and HepG2 cells transfected with different constructs. As shown in Fig.3A, PTB-U-box led to a significantly reduced cell growth from the time point of 4 days upon transfection. In contrast, PTB and the U-box mutant did not exhibit cell growth inhibition. Similar result was observed in HepG2 cells, showing an obvious growth inhibition in PTB-U-box transfectant compared with vector control (Fig. 3B). Further, The colony-formation assay showed that PTB-U-box expression markedly inhibited colony formation in both HeLa and HepG2 cells, whereas PTB and the PTB-U-box(HQ) transfectants revealed no significant effects (Fig. 3C and). Together, these data suggested that PTB-U-box could inhibit cell proliferation.

Furthermore, we determined whether PTB-U-box affects cell invasion by performing cell invasion assay. As shown in Fig.3E, the invasive cells of PTB-U-box transfectant were significantly less than those of vector control. However, PTB and PTB- U-box(HQ) transfection have no significant effect on cell invasion. Likewise, similar results were obtained in HeLa cells (Fig.3F). Collectively, these data indicated that PTB-U-box led to an inhibition of cancer cell invasion via down-regulating IGF-1R and IR.

### PTB-U-box inhibits activation of Akt and ERK signaling pathways and enhances chemo-sensitivity in cancer cells

**Figure 4:**
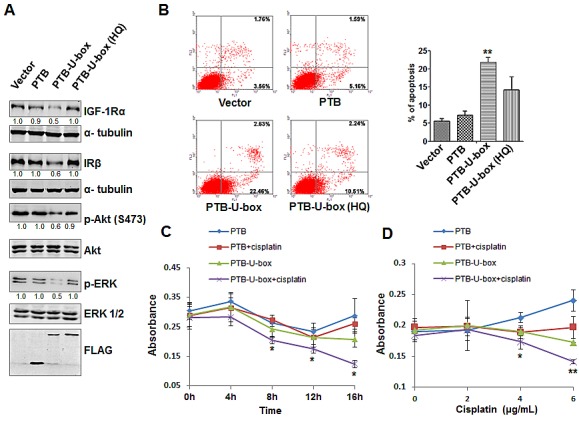
Targeted degradation of IGF-1R and IR attenuates Akt and MAPK signaling and increases the chemo-sensitivity of HepG2 cells. A, HepG2 cells transfected with indicated plasmids were lysed and subjected to Western blotting with the indicated antibodies, respectively. The bands intensity was quantified. B, HepG2 cells transfected with indicated plasmids were treated with cisplatin (3μg/mL) and cell apoptosis determined by Annexin V-PI stain and flow cytometry. The histogram represents the percentage of apoptotic cells. **P<0.01 for PTB-U-box *v.s.* vector. C and D, HepG2 cells transfected with indicated plasmids were treated with cisplatin (3μg/ml) for the indicated time interval (C), or with indicated concentration of cisplatin for 8h (D). Cell viability was measured by the MTT assay. *P<0.05 and **P<0.01 for PTB-U-box-transfection plus cisplatin *v.s.* PTB-U-box-transfection.

Activated IGF-1R and IR, through recruiting IRS-1 and other adaptors, is able to activate the downstream Akt and MAPK signaling pathway, both of which play important roles in cell proliferation, migration and survival [8-10]. Therefore, we assumed that down-regulation of IGF-1R and IR due to PTB-U-box expression also attenuate Akt and ERK activation. Indeed, as shown in Fig.4A, PTB-U-box transfection resulted in IGF-1R and IR down-regulation, accompanied by reduced phosphorylation of Akt and ERK, whereas the total Akt and ERK levels were not affected. In contrast, the expression of ligase activity-deficient forms, either PTB or PTB-U-box(HQ), did not alter phospho-Akt or phospho-ERK levels. Therefore, the targeted degradation of IGF-1R and IR effectively attenuates the downstream MAPK and PI3K/Akt signaling pathway.

Chemotherapy is a common therapeutic approach for several types of cancers. Cisplatin, a widely used chemotherapeutic agent, by binding to and causing crosslink of DNA, can trigger cell apoptosis [34], and thus exert its anti-tumor function. It has been shown that knockdown of IGF-1R enhances chemo-sensitivity of many types of cancers, such as liver cancer cells [31]. Therefore, we determined whether PTB-U-box-driven down-regulation of IGF-1R and IR also enhance the chemo-sensitivity. To this end, we first detected cisplatin-triggered apoptosis of HepG2 cells that were transfected with different constructs. As shown in Fig.4B, PTB-U-box-transfected HepG2 cells exhibited much higher cell apoptosis (21.85±1.33%) upon cisplatin treatment than the control (5.57±0.67%), PTB (7.2±1.15%) and PTB-U-box (H260Q) transfectants (14.23±3.58%). The similar results were obtained by using another chemotherapeutic agent, doxorubicin (Supplementary figure 5), suggesting that PTB-U-box indeed can enhance chemo-sensitivity. To further confirm this conclusion, PTB and PTB-U-box transfectants were untreated or treated with cisplatin of different doses or for different time interval, then the cell viability was evaluated by MTT assay. As shown in Fig.4C, in PTB-U-box-expressing HepG2 cells, low dose of cisplatin (3μg/mL) significantly and dramatically inhibits cell viability upon treatment for 8 hours. However, such dose of cisplatin has no significant effect on cell viability in PTB-expressing cells, even after incubation for 16 hours. Further, using different doses of cisplatin (2-6 μg/mL) to treat the cells for 8 hours, it was shown that cisplatin cooperates with PTB-U-box, rather than PTB, to promote cell death at concentration of 4-6 μg/mL (Fig.4D). Together, these data suggested that PTB-U-box could enhance chemo-sensitivity by down-regulating IGF-1R and IR protein levels and the downstream signaling.

### PTB-U-box inhibits translocation of Glut4 to membrane and glucose metabolism in HepG2 cells

**Figure 5:**
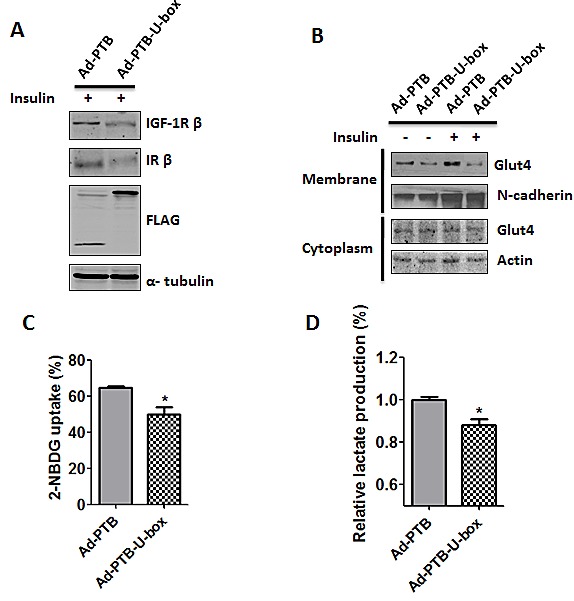
Ad-PTB-U-box inhibits Glut4 membrane translocation and glucose metabolism A, HepG2 were infected with Ad-PTB or Ad-PTB-U-box adenovirus (1×10^9^v.p.), and cell lysates were subjected to Western blotting analysis. B, HepG2 cells infected with the indicated adenovirus were treated with or without insulin. The membrane and cytoplasmic proteins were extracted and subjected to Western blotting with the indicated antibodies. C, Cells infected with the indicated adenovirus were cultured in the presence of the fluorescent glucose analog, 2-NBDG. Glucose uptake was quantified using FACS analysis. D, Cells were infected with the indicated adenovirus and lactate production was measured. *P<0.05 for Ad-PTB-U-box infection *v.s.* Ad-PTB infection.

As well known, increased glucose uptake and aerobic glycolysis is one of the critical hallmarks of cancer cells [35]. Upon activation, both IR and IGF-1R signal through Akt signaling pathway and lead to a various of downstream cellular effects, among which the translocation of glucose transporter 4 (Glut4) from cytoplasm to membrane will induce glucose uptake [36]. Therefore, we examined whether down-regulation of IR caused by PTB-U-box could also inhibit Glut4 translocation and glucose uptake in cancer cells. To this end, HepG2 cells were infected with the recombinant adenovirus which carries FLAG-tagged PTB-U-box (Ad-PTB-U-box) or PTB (Ad-PTB). The expression and down-regulatory effect of PTB-U-box on IGF-1R and IR were confirmed by Western-blot (Fig.5A). As shown in Fig.5B, membrane Glut4 but not cytoplasm Glut4 was significantly increased by insulin treatment in Ad-PTB infected cells. However, such an effect was almost abrogated in Ad-PTB-U-box infected cells. This was further confirmed by immunofluorescence staining, showing that insulin treatment led to more Glut4 translocation into the membrane in Ad-PTB but not Ad-PTB-U-box infected cells (Supplementary figure 6). As a result, glucose uptake (Fig.5C) as well as lactate production (Fig.5D) in Ad-PTB-U-box infected HepG2 cells was attenuated compared with Ad-PTB infected cells. Together, these results suggested that engineered ubiquitin ligase PTB-U-box, via IGF-1R and IR degradation, could inhibit cancer glucose metabolism through preventing Glut4 membrane translocation.

### PTB-U-box-carrying adenovirus suppresses HepG2 xenograft growth in mice

**Figure 6:**
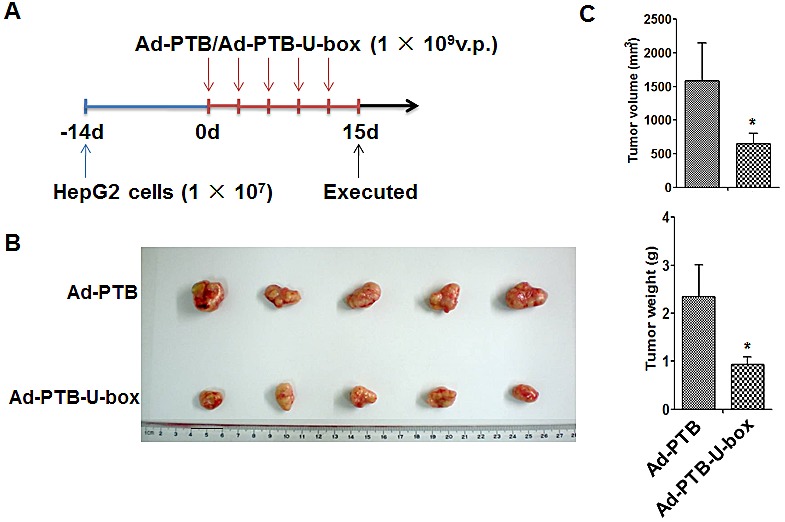
Ad-PTB-U-box exhibits potent anti-tumor effect in HepG2 xenograft A, Schematic representation of the process for HepG2-xenograft establishment and adenovirus treatment. The adenoviruses carrying PTB or PTB-U-box were intratumorally injected into the subcutenously inoculated tumors. B, The tumor nodules were collected at the end of treatment and photographed. C, The final tumor volume (mm^3^) was calculated by the formula (width^2^×length×0.5) (upper) and the tumor weight (g) was recorded (lower). Results are represented as the mean ± s.d. *P<0.05 for Ad-PTB-U-box injection *v.s.*Ad-PTB injection.

Based on the strong *in vitro* inhibitory effect of PTB-U-box on cell malignancy, we finally pursued the potential efficacy of Ad-PTB-U-box as an anti-tumor agent in HepG2-transplanted nude mice model. HepG2 xenograft were generated by subcutaneously injecting HepG2 cells into the right armpit of each mouse, and 2 weeks later, an intratumoral injection of Ad-PTB or Ad-PTB-U-box were performed repeatedly for up to 5 times (Fig.6A). At the end of the experiment, the mice were sacrificed and the resultant tumors were evaluated. As shown in Fig.6B and 6C, the average tumor volume and tumor weight of Ad-PTB-U-box-treated mice were significantly less than those of Ad-PTB-treated mice (P<0.05). Collectively, these data indicated that the targeted degradation of IGF-1R/IR by Ad-PTB-U-box significantly inhibit the *in vivo* tumor growth of IGF-1R/IR-overexpressing cancer cells, implying its potential as a therapeutic agent.

## DISCUSSION

Although IGF-1R and IR are both widely expressed on normal tissues, the strong implications of IGF-1R in various cancers have already been well recognized and therapeutic strategies with antibodies and small molecule TKIs have been actively tested in clinical trials [1, 2, 4, 37-39]. On the other hand, however, the oncogenic role of IR and consideration of IR-targeted strategy has attracted much attention most recently. Using xenograft and transgenic mouse model, it was shown that IR knockout or knockdown inhibits tumorigenesis, angiogenesis and metastasis [11, 18, 33, 40]. In addition, there is compelling evidence that compensatory crosstalk between IGF-1R and IR accounts for resistance to anti-IGF-1R therapy [18, 41]. All of these findings provide the rationale for co-targeting IGF-1R and IR in cancer treatment. Actually, dual IGF-1R/IR tyrosine kinase inhibitor, such as OSI-906, has already been developed, with some of them being taken into clinical trials [42]. It has also been shown that OSI-906 provides superior anti-tumor efficacy compared with targeting anti-IGF-1R antibody alone [41]. Our study hereby provides another alternative strategy -- targeted degradation of IGF-1R and IR simultaneously -- for the treatment of cancers with IGF-1R/IR co-expression and over-activation.

Targeted degradation of a specific protein by harnessing the endogenous ubiquitin system represents an alternative approach that knocks down the target protein at protein levels, also known as “protein knockout” [43-45]. This can be achieved by creating either an artificial E3 ubiquitin ligase that recognizes proteins of interest, or a small “adaptor” molecule that can bridge the target proteins to an E3 ubiquitin ligase, ultimately resulting in ubiquitination-mediated proteolysis. Over the past decade, several groups have successfully utilized such approaches to eradicate the disease-associated proteins [43]. On the other hand, different chimeric E3 ubiquitin ligases, by fusing an “interacting domain” with an “E3 catalytic domain”, have been reported to specifically degrade cancer-causing proteins, including β-catenin [46], KRAS [47] and ErbB1-3 [44], thus successfully inhibiting the related tumor growth in cultured cells and animal models. Our study presented here further strengthens the proof-of-concept of targeted ubiquitination and degradation of oncogenic RTKs. Together with previous studies from ours [48] and others [44], these data imply that rewiring oncogenic RTKs to degradation by recombinant ubiquitin ligase represents an effective therapeutic strategy for the treatment of cancers with aberrant RTKs.

There are several obvious advantages of the targeted protein degradation for cancer treatment. First, because target proteins are eliminated directly in protein level, it can overcome anti-cancer agents-induced secondary mutations, a main mechanism for acquired drug resistance. Second, well-designed engineered ubiquitin ligases are able to degrade multiple oncoproteins which share the same binding partner, thereby achieving the goal of combinatory therapy. This is of great importance because most type of cancer cells either over-express more than one oncoproteins simultaneously or they can activate the compensatory pro-survival pathway upon single target-directed therapy. Third, this RTKs-targeting degradation strategy can be combined with antibodies or small molecule inhibitors against RTKs, especially when kinase-independent biologic functions of RTKs, such as IGF-1R and EGFR, also contribute to cancer malignancy [49]. Finally, our strategy described here and previously [48] takes advantage of the functional E3 domain from mono-molecule E3 ligase, i.e., Cbl or CHIP, but not that from multi-subunit E3 complex such as Skp-Cullin-F-box complex, thus it does not necessitate the presence or abundance of the other components of E3 complex. Taken together, such a strategy may hold the promises for future cancer treatment and drug resistance.

In this study, we have created and screened several engineered ubiquitin ligases which are composed of an IGF-1R/IR-binding domain and a functional ubiquitin ligase domain. We identified that PTB-U-box was more potent as for degradation effect on the two target proteins compared with PTB-RING. It is possible that the structure of PTB-RING is not optimized for its E3 activity, or other regulations such as RING-mediated autoubiquitination exist and affect its function. Upon ectopic expression, PTB-U-box enhances IGF-1R/IR ubiquitination and degradation, thereby inhibiting several key cancer hallmarks, i.e., proliferation, invasion, drug insensitivity and glucose metabolism, and thus retarding the tumor growth in xenograft. However, the two E3 activity-defective constructs, PTB and PTB-U-box (HQ), have no inhibitory effect on cell malignancies, albeit they are capable of binding to IGF-1R/IR and supposed to interfere with the recruitment of IRS-1 to IGF-1R/IR and downstream signaling to some extent. We reason that, in situation of PTB/PTB-U-box(HQ) overexpression, the unchanged receptors may recruit other adaptors such as IRS-2-4 and thus compensate for IRS-1 interference, which is supported by unchanged Akt and ERK activation. Thus, the efficacy of PTB-U-box attributes to PTB-U-box-caused IGF-1R/IR degradation and subsequent downstream signaling inhibition. Our finding is consistent with the previous studies, showing that IGF-1R and/or IR knockdown inhibits the tumor growth as well as increases the chemo-sensitivity [31, 33, 50]. Notably, by targeting both IR and IGF-1R, PTB-U-box was able to suppress glucose metabolism of cancer cells. Indeed, the strategies that target the cancer metabolism are being actively tested recently [51], among which targeting insulin system such as insulin inhibition and metformin has shown promising anti-tumor effect. The advantage of co-targeting IR and IGF-1R also lies in simultaneous attenuation of cancer growth and cancer metabolism, which can strengthen the anti-tumor efficacy.

In this study, to evaluate the *in vivo* efficacy of PTB-U-box, we used adenovirus (Ad) as a delivery method. Adenovirus is one of the most frequently used vectors in cancer gene therapy because of several advantageous characteristics, including high gene transfer efficiency in both dividing and non-dividing cells, lack of insertional mutagenesis and induction of oncolysis by viral replication [52]. The limitations are their potential immunogenicity, transient expression and short half-life time in the target cells, especially for long-term and systemic administration. Encouragingly, however, such shortcomings might be overcome with the development of new Ad delivery systems. For example, modification of Ad with polymers or nanomaterials can minimize the immune response and degradation and improve the transduction efficacy; smart Ad/nanohybrid systems, through conjugating targeting ligands with Ad, can significantly increase the selectivity/specificity of cellular uptake, transgene expression levels and anti-tumor efficacy [52].

Another major concern for our strategy may be insulin resistance, which may result from PTB-U-box-trigged IR down-regulation, especially in the case of non-selective accumulation of adenoviruses in cancers and the adjacent normal tissues. However, such type of insulin resistance in normal cells, due to decreased IR and IGF-R signaling, could be beneficial, because the concomitant decrease in mTOR activity can slow down aging, prolong life span and prevent cancer [53, 54]. Particularly, recent studies favor the notion that attenuation of IGF-1 signaling can protect normal cells, but not cancer cells, against fasting and chemotherapy [55]. Moreover, it has been well documented that direct or indirect inhibition of insulin (or IGF-1)/PI3K/Akt/mTOR pathway, such as rapamycin, metformin and dietary protein restriction, have cancer- preventing or treating effects [54, 56]. These evidences, together with the improvement and optimization of Ad delivery system, highly support that systemic administration of our PTB-U-box adenovirus is feasible. In the future study, it merits to evaluate the therapeutic and preventative effect of PTB-U-box on cancers by using different types of orthotopic cancer models.

In summary, herein we artificially created an engineered ubiquitin ligase, which is composed of an IGF-1R/IR-binding domain and a functional E3 ubiquitin ligase domain, so as to target the ubiquitination and degradation of activated IGF-1R/IR. We have provided evidences that such an engineered molecule, PTB-U-box, can effectively degrades IGF-1R/IR, inhibits cell proliferation and invasion, increases chemo-sensitivity and reduces glucose metabolism when ectopically expressed in cancer cells. Moreover, intratumoral injection of adenovirus carrying PTB-U-box dramatically retards the growth of HepG2 xenograft. Therefore, co-targeting IGF-1R and IR for ubiquitination and degradation by well-designed engineered ubiquitin ligase represent an alternative therapeutic strategy for the treatment of cancers co-expressing IGF-1R/IR.

## METHODS

### Cell lines, antibodies and reagents

Human cervical cancer HeLa, liver cancer HepG2, pancreatic cancer PANC-1 and human embryonic kidney cell HEK-293T cell lines were maintained in DMEM (Life Technologies) supplemented with 10% fetal bovine serum. All these cell lines were cultured in a 37°C incubator with 5% CO2 humidified air. Antibodies against IGF-1Rα, IGF-1Rβ, IRβ,β-Actin and α-tubulin were obtained from Santa Cruz Biotechnology (Santa Cruz, CA, USA), FLAG and IgG were from Sigma-Aldrich (St Louis, MO, USA), and Glut4, Akt, p-Akt(S473), ERK and p-ERK from Cell Signaling (Andover, MA, USA). Antibody against HA was purchased from NeoMarkers (Fremont, CA, USA). Antibody against N-cadherin was from BD Biosciences. IGF-1 and insulin were obtained from Sigma-Aldrich, and the proteasome inhibitor MG132 from Calbiochem (Billerica, MA, USA). SYBR Green universal master mix and Multiscript RT were purchased from TaKaRa Biotechnology (Dalian) Co., Ltd (Dalian, China). Cy3- and FITC-conjugated secondary antibodies and 4’,6-diamidino-2-phenylindole (DAPI) were purchased from Boster Biology Company (Wuhan, China). 2-[N-(7-Nitrobenz-2-oxa-1,3-diazol-4-yl)amino]-2-deoxy-D-glucose (2-NBDG), a fluorescence labeled deoxyglucose analog, was obtained from Cayman Chemical Company (Ann Arbor, MI, USA).

### Plasmids construction and cell transfection

DNA segment encoding PTB domain was amplified from cDNA of HeLa cells. U-box domain was amplified from pcDNA3.0-CHIP, provided by W.P. Xu (National Cancer Institute, Rockville, MD), and RING domain was amplified from the pEFHACbl plasmid (a kind gift from Y.C. Liu, La Jolla Institute for Allergy and Immunology, La Jolla, CA). PTB-U-box and PTB-RING were generated by fusing PTB with U-box or RING, using overlapping extension PCR. PTB, PTB-U-box and PTB-RING were then cloned into *EcoR* I/*EcoR* V sites of the pFLAG-CMV-4 vector. PTB-U-box (H260Q) was amplified from pFLAG-CMV4-PTB-U-box by site-directed mutagenesis using PfuUltra™ High-Fidelity DNA Polymerase (stratagene, La Jolla, CA). pcDNA3.1(+)−3×HA-Ub were gifts from David Dornan (Genentech, Inc., South San Francisco, CA). pBABE-bleo IGF-1R was purchased from Addgene (Addgene plasmid 11212). All constructs were verified by DNA sequencing. For cell transfection, HeLa, HepG2, PANC-1 and HEK-293T cells were transfected with LipofectAMINE™2000 (Invitrogen) according to the manufacturer’s protocol.

### Quantitative real-time PCR (qRT-PCR) analysis

The cells was transiently transfected with the indicated plasmids and 48h later, total RNA was extracted using TRIZOL reagent (Invitrogen) according to the manufacturer’s protocol. The first strand cDNA was generated from total RNA (2μg) with reverse transcriptase (Promega, WI, USA) and used as the template for qRT-PCR analysis. GAPDH cDNA was used as an internal control to normalize variances. The primers used were as follows: IGF-1R, 5’-TCTGGCTTGATTGGTCTGGC-3’ (forward), 5’-aaccattggctgtgcagtca-3’ (reverse); IR, 5’-gcctctacaacctgatgaac-3’ (forward), 5’-acagatgtctccacactcc-3’ (reverse); GAPDH, 5’-ctgcaccaccaact gcttag-3’ (forward), 5’-ttctgggtggcagtgatg-3’ (reverse). PCR was performed in Prism 7500 real-time thermocycler (ABI). PCR conditions were 30s at 95°C, followed by 10s at 95°C and 30s at 60°C for 40 cycles.

### Co-immunoprecipitation and Western blotting

For co-immunoprecipitation assay, cells transfected with the indicated plasmids were starved for 12 hours and 15min before collection, IGF-1 (100ng/ml) or insulin (1μg/ml) were added into the medium. Then the cells were lysed and the lysates containing 1 to 1.5 mg total proteins were incubated with anti-IGF-1Rα/β or anti-IRβ antibodies for 3 h at 4°C, followed by incubation with protein A Sepharose beads over night at 4°C. The precipitates were resolved by 8% to 15% SDS-PAGE and transferred to nitrocellulose membranes. For *in vivo* ubiquitination assay, cells transfected as indicated were starved for 12 hours and then treated with 10μM MG-132 for 4 h and with insulin (1μg/ml) or IGF-1(100ng/ml) for 15 min before harvesting. For Western blotting, cell extracts containing 30 to 50 μg total protein were directly subjected to SDS-PAGE and transferred. The membranes were blocked with 5% BSA or 5% milk, probed with indicated primary antibodies and the corresponding secondary antibodies, and then detected by enhanced chemiluminescence or by using the Odyssey Imaging System (Li-Cor Biosciences). The bands intensity were quantified by densitometry and normalized to α-tubulin using Image J analysis software.

### Cycloheximide (CHX) chase experiment

HeLa cells were transfected with the indicated plasmids and twenty-four hours later, the cells were treated with CHX (50μg/ml) for 0, 6, 12 and 24 hours. Then the cells were harvested and cell lysates were subjected to Western blotting analysis to assess the protein stability of IGF-1R and IR.

### Cell growth, colony formation, and cell invasion assays

For cell growth assays, twenty-four hours after transfection, cells were seeded at 2,000/well in septuple in 96-well. Cell growth was assessed using 3-(4,5-dimethylthiazol-2-yl)-2,5-diphenyltetrazolium (MTT) assay every day.

For colony formation assays, cells transfected with the indicated plasmids and seeded at 200/well in six-well plates in triplicate and cultured for 14-21 days using the complete medium containing G418 (600-800μg/mL). Cell colonies were fixed and then stained with Giemsa for 20 to 30 min. The number of colonies was reported, and the colony formation ratio was calculated according to the following formula: colony formation ratio (%) = (colony number/seeded cells number) ×100%.

Cell invasion assay was performed in matrigel-coated transwell chambers (8-μm pore size, BD Pharmingen). Cells transfected with the indicated plasmids were seeded in triplicate at 2×10^4^ in 0.1% FBS containing medium per upper chamber, which were placed in 24-well tissue culture plates containing 5% FBS containing medium. Twenty-four hours later, the invasive cells was stained with Giemsa and photographed under the microscope. The amount of invasive cells for each group was calculated in ten random fields (400×magnification) and the data were reported as the mean±s.d.

The above experiments were repeated at least in triplicate.

### Drug sensitivity assay

Cells were seeded in 6-well plate in triplicate and transfected with indicated plasmids, and 48 h later, treated with 3μg/ml cisplatin (or 0.5μg/ml doxorubicin) for 8 h. 1×10^5^ cells were collected, doubly stained with propidium iodide (PI) and Annexin V, and cell apoptosis were measured by flow cytometry. Or, cells were seeded in 96-well plate in sextuple and transfected with the indicated plasmids. Forty-eight hours later, cells were treated with 3μg/ml cisplatin for the indicated time interval or with the indicated concentration of cisplatin for 8h, and cell viability assessed by MTT assay. Each experiment was conducted in triplicate.

### PTB/PTB-U-box recombinant adenovirus generation and cell infection

The recombinant adenovirus carrying FLAG-tagged PTB or PTB-U-box (Ad-PTB or Ad-PTB-U-box) was generated and provided by Vector Gene Technology Company Ltd (Beijing, China). The final titer of the purified recombinant adenovirus was 1×10^11^v.p./mL. For *in vitro* cell infection, HepG2 was seeded at 1×10^5^ in 6-well plates and infected with 1×10^9^ v.p. recombinant adenovirus. Forty-eight hours later, the cells were collected and cell lysates were subjected to Western blot analysis, or the cells were subjected to the analysis described below.

### Glucose uptake assay

HepG2 cells seeded in 6-well plate in triplicate were infected with Ad-PTB or Ad-PTB-U-box adenovirus respectively. Thirty-six hours later, cells were serum-starved (0.1% FBS) for 12 hours, refreshed with glucose-free DMEM for 2 hours, and then 100μM 2-NBDG was added into the cell medium for a duration of 30 min. Then the cells were treated with or without insulin (1μg/ml) for another 15 min, and glucose uptake was quantified using FACS analysis. The experiment was repeated in triplicate.

### Lactate production assay

HepG2 cells were seeded in 24-well plate (3×10^4^) in triplicate and infected with Ad-PTB or Ad-PTB-U-box adenovirus respectively. Thirty-six hours later, culture medium was removed from cells and lactate concentration was determined using lactate test kits (Nanjing Jiancheng Bioengineering Institute, Nanjing, China) according to the manufacturer’s instruction. Next, cells were harvested and cell numbers were counted directly under the microscope using hemocytometer. Last, the rate of lactate production were determined (lactate production rate = lactate concentration/cells/time). The experiment was repeated in triplicate.

### Anti-tumor effect of Ad-PTB-U-box in HepG2 xenograft

HepG2 xenograft was established by subcutaneously injecting HepG2 cells into the right armpits of six-week-old BALB/c nude mouse (1×10^7^ cells/per mouse; five animals per group). Two weeks later, when the tumor is visible, mice were treated with an intratumoral (IT) injection of 100μl adenovirus (1×10^10^ v.p./mL) of Ad-PTB or Ad-PTB-U-box once every 3 days for up to 5 times. At the end of the experiment, tumor growth was monitored by measuring tumor size and calculating tumor volume using a standard formula: tumor volume (mm^3^) = width (mm)^2^×length (mm)×0.5. Mice were then sacrificed, the tumors were isolated, and tumor weight assessed.

### Statistical analysis

Statistical analysis was performed with the SPSS17.0 software package for Windows by using the two-sided Student’s t-test for independent groups. Statistical significance was based on a value of P < 0.05. Data are expressed as mean±s.d.

## SUPPLEMENTARY FIGURES


