# Supplementary material for: Engineered ubiquitin ligase PTB-U-box targets insulin/insulin-like growth factor receptor for degradation and coordinately inhibits cancer malignancy
Source: Oncotarget. 2014 Jun 6;5(13):4945–58. doi: 10.18632/oncotarget.2066 (PMC4148113; doi:10.18632/oncotarget.2066)
Supplement: Supplementary file 1 [file oncotarget-05-4945-s001.pdf]

Engineered ubiquitin ligase PTB-U-box targets insulin/insulin-like growth factor receptor for degradation and coordinately inhibits cancer malignancy

Supplementary methods and Material

Detection of IGF-1Rβ and IRβ phosphorylation by co-immunoprecipitation

HepG2 cells were starved for 12 hours and 15min before collection, IGF-1 (100ng/ml), insulin (1μg/ml) or fetal bovine serum were added into the medium. Then the cells were lysed and the lysates containing 1 to 1.5 mg total proteins were incubated with anti-IGF-1Rβ or anti-IRβ antibodies for 3 h at 4°C, followed by incubation with protein A Sepharose beads over night at 4°C. The precipitates were resolved by 8% SDS-PAGE and transferred to nitrocellulose membranes. The membranes were blocked with 5% BSA or 5% milk, probed with anti-pTyr antibody (4G10), anti-IGF-1Rβ or anti-IRβ antibodies respectively. After incubation with the corresponding secondary antibodies, the signal were detected by using the Odyssey Imaging System (Li-Cor Biosciences).

Immunofluorescence staining and confocal microscopy analysis of Glut4

HepG2 cells were infected with Ad-PTB or Ad-PTB-U-box adenovirus and 48 hours later, treated with or without insulin (1μg/ml) for 15min, then the cells were fixed with 4% Paraformaldehyde (PFA) for 15 min at room temperature and blocked with 5% BSA in PBS for 2 hr. Cells were incubated with the rabbit anti-Glut4 antibody and the mouse anti-FLAG antibody respectively, followed by incubation with the Cy3-conjugated goat-anti-rabbit and FITC-conjugated goat-anti-mouse secondary antibodies. Cells were then incubated with DAPI (0.25mg/ml) for 10 min to stain cell nuclei. The cells were detected with laser confocal microscopy and the merged photographs were taken.

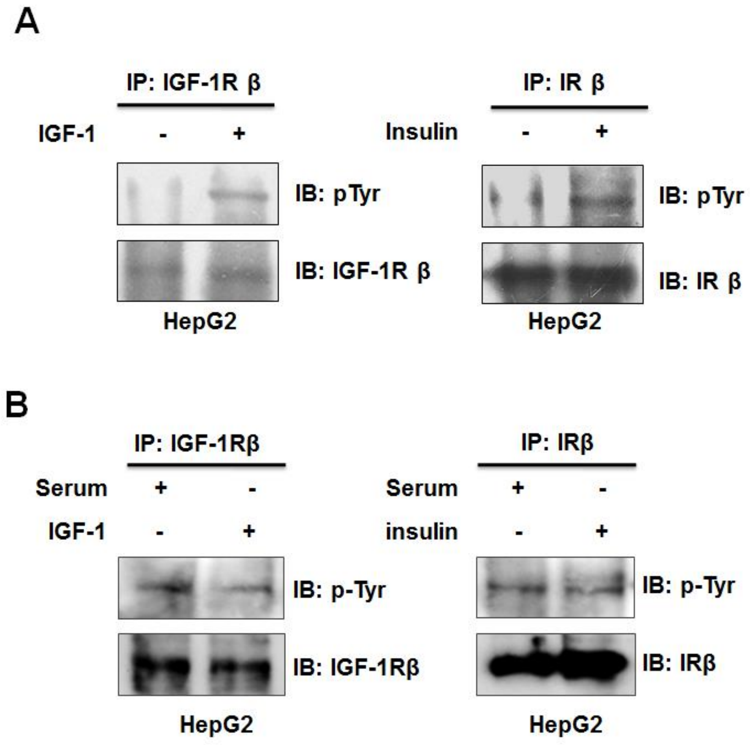

Supplementary figure S1: IGF-1, inuslin or serum stimulation promotes phosphorylation of β subunit of IGF-1R and insulin receptor. HepG2 cells were starved for 12 hours, treated with insulin, IGF-1 (A and B) or 10% fetal calf serum (referred as serum) (B) for 15min and then lysed. Phosphorylation of IRβ and IGF-1Rβ were analyzed by immunoprecipitation with anti-IGF-1Rβ or anti-IRβ followed by Western blotting with 4G10 antibody.

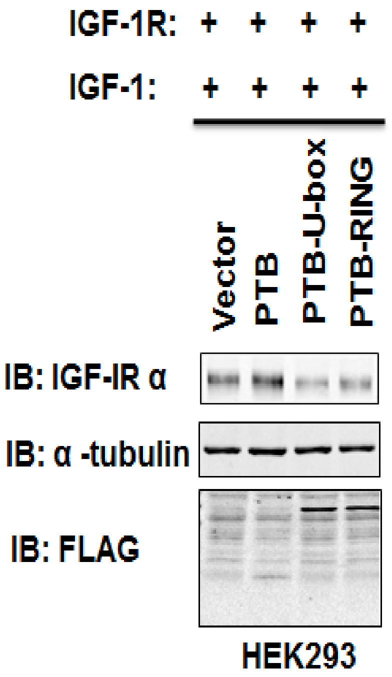

Supplementary figureS2: PTB-U-box and PTB-RING down-regulate exogenous IGF-1R in HEK-293 cells. HEK-293 cells were transiently transfected with IGF-1R encoding plasmid and 48 hours later, cells were serum starved for 12 h and treated with IGF-1 for 15min. Cell lysates were subjected to Western blotting with anti-IGF-1Rα, anti-α-tubulin and anti-FLAG antibodies.

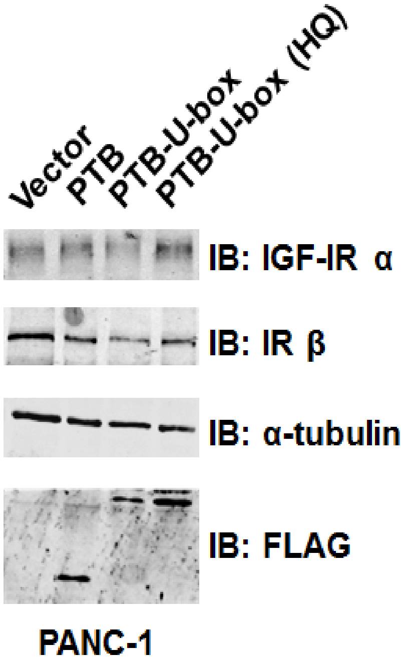

Supplementary figure S3: PTB-U-box causes IGF-1R and IR down-regulation in PANC-1 cells. PANC-1 cells transiently transfected with the indicated constructs were lysed and analyzed for down-regulation of IGF-1R and IR by Western blotting.

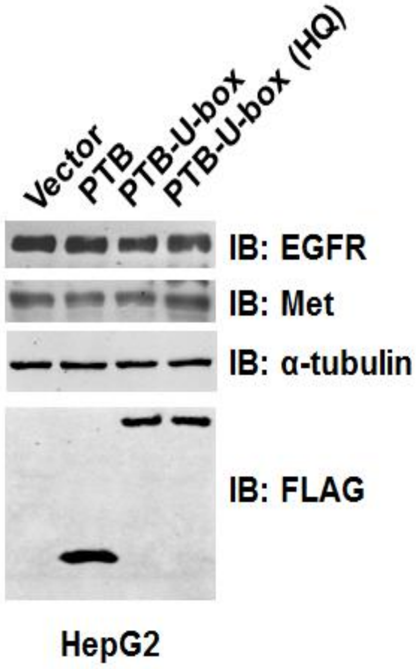

Supplementary figure S4: PTB-U-box does not down-regulate EGFR and Met protein levels. HepG2 cells were transiently transfected with the indicated constructs and protein levels of EGFR and Met were analyzed by Western blotting.

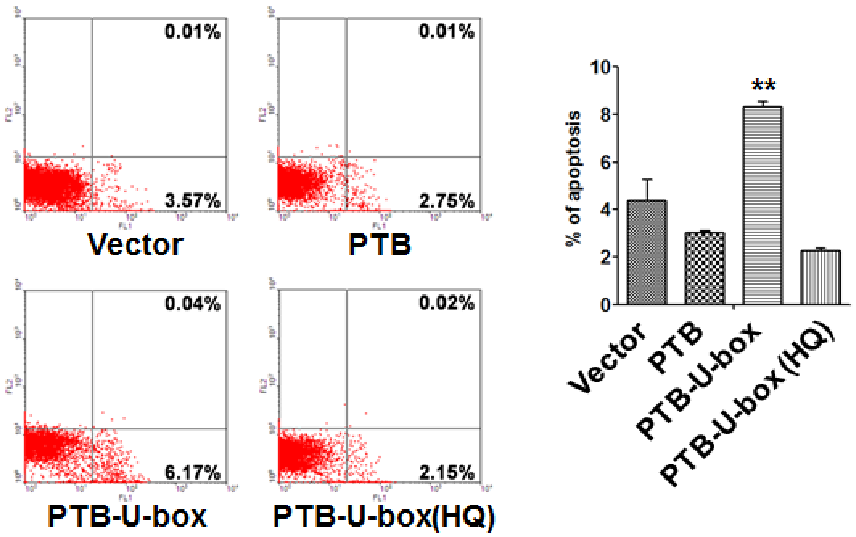

Supplementary figure S5: PTB-U-box enhances sensitivity of HepG2 cells to doxorubicin-induced apoptosis. HepG2 cells transfected with indicated plasmids were treated with doxorubicin (0.5μg/mL) for 8h and cell apoptosis were determined by Annexin V-PI flow cytometry analysis. The histogram represents the percentage of apoptotic cell proportion. Control (4.36±1.54%), PTB (3.03±0.08%), PTB-U-box (8.33±0.38%), PTB-U-box(HQ) (2.25±0.21%). ** P<0.01 for PTB-U-box *v.s.* vector.

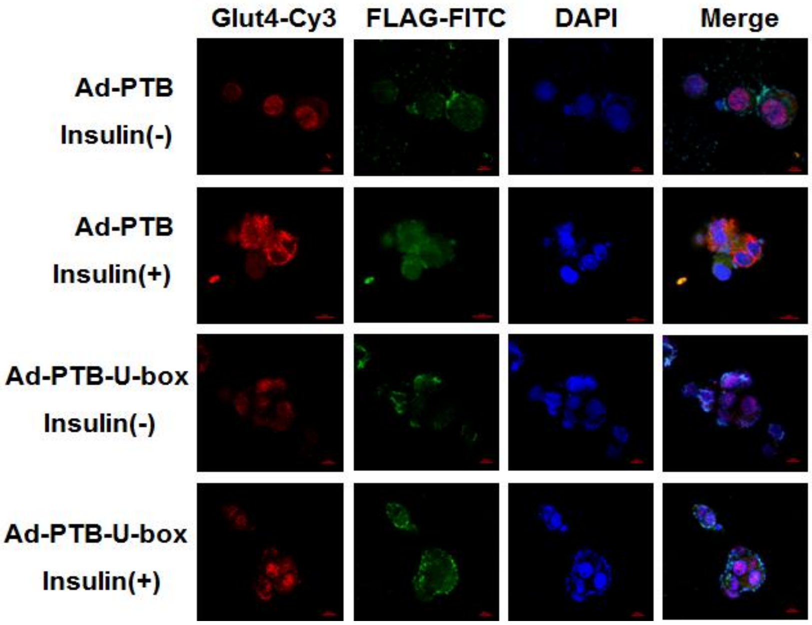

Supplementary figure S6: Ad-PTB-U-box inhibits Glut4 membrane translocation. HepG2 cells infected with the indicated adenovirus were treated with or without insulin. The cells were subjected to immunofluorescent staining with anti-Glut4 and anti-FLAG antibodies, and detected by laser scanning confocal microscope. Bar:20 μm.
